# EGF-Mediated Overexpression of Myc Attenuates miR-26b by Recruiting HDAC3 to Induce Epithelial-Mesenchymal Transition of Lens Epithelial Cells

**DOI:** 10.1155/2018/7148023

**Published:** 2018-05-30

**Authors:** Ning Dong, Bing Xu, Jingmei Xu

**Affiliations:** Department of Ophthalmology, Beijing Shijitan Hospital, Capital Medical University, Beijing, China

## Abstract

The previous study has demonstrated that epidermal growth factor (EGF) and EGF receptor (EGFR) signaling plays a critical role in the development of posterior capsule opacification (PCO) through regulating lens epithelial cells (LECs) proliferation. Recent studies have suggested that the residual LECs undergo proliferation and migration, and epithelial-mesenchymal transition (EMT) is the important cause of PCO formation after cataract surgery. EMT of LECs is considered to be playing a central role in the pathogenesis of PCO. In the present study, we investigated whether and how EGF may regulate EMT of LECs. First, we demonstrated that EGF and EGFR signaling induces Myc overexpression in primary human lens epithelial cells (HLECs). In turn, Myc overexpression could inhibit miR-26b by recruitment of HDAC3. Consequently, the downregulated expression of miR-26b increased the expression of EZH2 in primary HLECs. Mechanistically, miR-26b directly controls EZH2 expression by targeting its 3′-UTR in HLECs by luciferase reporter assays. Finally, we demonstrated that EGF induces the expression of EMT markers in primary HLECs via a miR-26b-dependent mechanism. In summary, EGF activated Myc and Myc overexpression inhibited miR-26b by recruitment of HDAC3, which in turn induced the expression of EZH2 and promoted the progression of EMT in HLECs.

## 1. Introduction

After cataract operation, residual lens epithelial cells (LECs) initiate a wound-healing response, which leads to impairment of vision [1, 2]. It is known as posterior capsule opacification (PCO), also known as secondary cataract [1, 2]. Following cataract surgery, the residual LECs undergo proliferation and migration, and epithelial-mesenchymal transition (EMT) is the important cause of PCO formation [1–7]. EMT of LECs is considered to be playing a central role in the pathogenesis of PCO. EMT results in loss of lens epithelial cells adhesion and apical-basal polarity, and LECs also transdifferentiate into mesenchyme-like cells. During EMT, LECs undergo downregulation of epithelial differentiation markers, such as E-cadherin, and LECs acquire mesenchymal markers, such as fibronectin and alpha-smooth muscle actin (*α*-SMA) [3–6].

Recent studies have suggested that many growth factors in the aqueous humor, such as epidermal growth factor (EGF), hepatocyte growth factor (HGF), and transforming growth factor *β* (TGF-*β*), increase after cataract surgery [2, 3]. These growth factors are recognized to play a critical role in the development of PCO [2, 8]. Earlier studies have shown that EGF is involved in regulating cell proliferation through activating the EGF receptor (EGFR) signaling pathway. The previous study has demonstrated that the repression of EGFR can effectively prevent the progression of PCO by inhibition of LECs proliferation [3]. Recently, EGF has been associated with the initiation of EMT in many cancers [9]. Whether EGF and EGFR signaling can regulate EMT of LECs and be involved in the development of PCO is an important area of research.

Here, we present the evidence confirming that EGF and EGFR signaling induces the expression of Myc in the LECs. Myc overexpression inhibits miR-26b by recruitment of HDAC3, which in turn induces the expression of enhancer of zeste homolog 2 (EZH2) and promotes the progression of EMT.

## 2. Materials and Methods

### 2.1. Patient Lens Epithelial Cell Collection and Culture

Patient LECs collection and culture were performed as described previously [5, 6]. Briefly, fresh lens capsules with adherent LECs were obtained from the Department of Ophthalmology, Beijing Shijitan Hospital, Capital Medical University (Beijing, China), during cataract surgery from 62 eyes with the clinical diagnosis of nuclear or anterior polar cataracts. The ages of the patients ranged from 58 to 72 years. The study was approved by the Ethics Committee of Beijing Shijitan Hospital and was performed in accordance with the Declaration of Helsinki. Each subject received a detailed information leaflet and provided informed written consent before participation. Fresh PCO tissues and normal attached LEC samples from organ donors were provided by the Eye Bank of Beijing, China (Beijing, China).

Primary human lens epithelial cells (HLECs) were cultured as described previously [6]. Primary HLECs were used to determine the role of EGF and EGFR signaling in EMT of LECs.

### 2.2. SRA01/04 Cell Culture

The human lens epithelial cell line SRA01/04 was cultured as described previously [5, 6]. SRA01/04 cell line was obtained from the Cancer Institute and Hospital, Chinese Academy of Medical Sciences (Beijing, China).

SRA01/04 cells were only used for the luciferase study.

### 2.3. RNA Interference

RNA interference was performed as described previously [5, 6, 10–12]. According to the manufacturer's instructions, EGFR siRNAs, Myc siRNAs, and EZH2 siRNAs (Cell Signaling Technology, Beverly, MA, USA) or HDAC1~HDAC11 siRNAs (Santa Cruz Biotech, Santa Cruz, CA, USA) were complexed with Lipofectamine™ 2000 (Invitrogen, Carlsbad, CA, USA) in 6-well plates. 2 *μ*l Lipofectamine 2000 was diluted in 50 *μ*l Dulbecco's Modified Eagle's Medium/Ham's Nutrient Mixture F-12 (DMEM/F12) (Sigma, St. Louis, MO). Then, 0.01 to 0.20 *μ*g siRNA was combined with transfection reagent after 15-minute incubation at room temperature. Next, the transfection was continued for 24 h at room temperature and underwent further experimentation.

### 2.4. Transfection

According to the manufacturer's instructions of GenePORTER transfection reagent (GTS Inc., San Diego, CA), primary HLECs were transiently transfected with 100 nmol/l miR-26b mimics (GenePharma, Shanghai, China) or anti-miR-26b (GenePharma) or miR-26b mimics negative control (GenePharma) or anti-miR-26b negative control (GenePharma). After 6 h, the supernatant was removed, and fresh medium was added.

### 2.5. Quantitative Reverse Transcription-PCR (qRT-PCR)

qRT-PCR was performed as described previously [5, 6, 10–12]. Small nuclear RNA (snRNA) U6 as the normalization control was used for qRT-PCR of miR-26b. Furthermore, glyceraldehyde 3-phosphate dehydrogenase (GAPDH) as the normalization control was used for qRT-PCR of fibronectin, *α*-SMA, Myc, EZH2, and HDAC1~11. qRT-PCR primers are shown in Table 1.

### 2.6. Western Blot Analysis

The Western blot analysis was performed according to the methods described previously [5, 6, 10–12]. The primary antibodies were from Abcam (Cambridge, MA, USA), including anti-EGFR, anti-Myc, anti-EZH2, anti-E-cadherin, anti-fibronectin, and anti-actin antibodies.

### 2.7. Luciferase Assay

Luciferase assay was performed as described previously [5, 6, 11, 12]. The 3′-UTRs of EZH2 containing the predicted miR-26b binding or mutant sites were amplified by PCR. The primers are shown in Table 1.

### 2.8. Statistical Analysis

All experiments were repeated at least three times. All data are presented as the mean ± SE. The statistical analyses were performed using the program SPSS for Windows Version 17.0 (SPSS, Inc., Chicago, IL, USA). Differences among experimental groups were evaluated by one-way analysis of variance (ANOVA) or Student's *t*-test. *P* values of 0.05 or less were considered statistically significant.

## 3. Results

### 3.1. EGF Induces the Expression of EMT Markers in Primary HLECs

**Figure 1:**
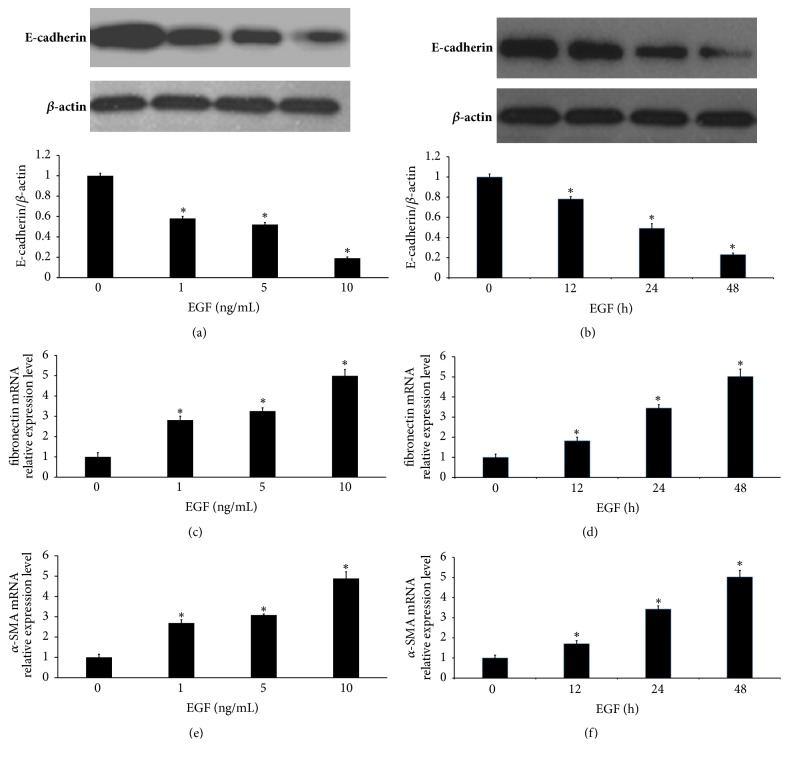
EGF induces EMT in primary HLECs. (a) The primary HLECs were treated with different concentrations of EGF for 48 h. The expression of E-cadherin proteins in primary HLECs was determined by Western blot. (b) The primary HLECs were treated with 10 ng/mL of EGF for different time. The expression of E-cadherin proteins was measured by Western blot. (c–f) The expression of fibronectin mRNA and *α*-SMA mRNA was measured by qRT-PCR. (c, e) The primary HLECs were treated with different concentrations of EGF for 48 h. (d, f) The primary HLECs were treated with 10 ng/mL of EGF for different time. ^*∗*^*P* < 0.05 compared with group without EGF.

Increasing evidence indicates that LECs undergo downregulation of epithelial differentiation markers, such as E-cadherin, during EMT [3–6]. To investigate the effect of EGF-induced EMT in primary HLECs, the LECs were treated with EGF. As shown in Figures 1(a) and 1(b), EGF suppressed E-cadherin expression in a dose-dependent manner and a time-dependent manner in primary HLECs. In parallel, EGF induced a dose-dependent and a time-dependent increase of the expression of fibronectin and *α*-SMA, which were mesenchymal-related markers (Figures 1(c)–1(f)).

### 3.2. Expression of Myc and EZH2 Is Increased in Human PCO Attached LECs and LECs Obtained from Patients with Anterior Polar Cataracts

**Figure 2:**
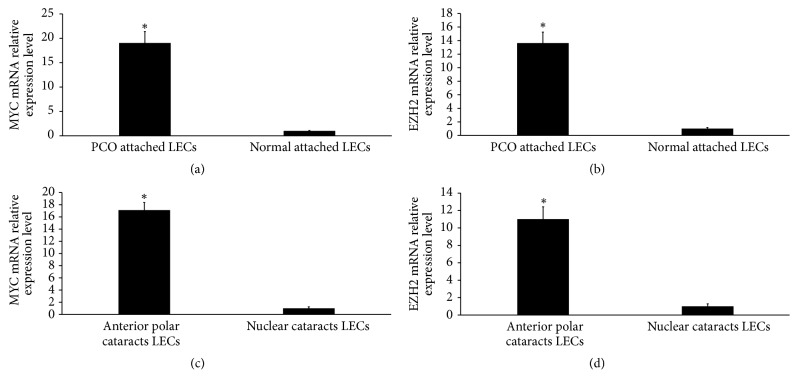
The expression levels of MYC mRNA and EZH2 mRNA are increased in human PCO attached LECs and LECs obtained from patients with anterior polar cataracts. ((a) and (b)) Upregulation of MYC mRNA and EZH2 mRNA was detected in human PCO attached LECs compared with normal attached LECs by qRT-PCR. ((c) and (d)) Upregulation of MYC mRNA and EZH2 mRNA was detected in the LECs obtained from patients with anterior polar cataracts compared with patients with nuclear cataracts by qRT-PCR. ^*∗*^*P* < 0.05 compared with normal-attached LECs ((a) and (b)). ^*∗*^*P* < 0.05 compared with nuclear cataracts LECs ((c) and (d)).

It is generally acknowledged that LECs can transdifferentiate and proliferate into mesenchyme-like cells or myofibroblasts and undergo EMT during the formation of anterior polar cataracts [13–15]. Our previous study has demonstrated that the expression of E-cadherin was downregulated and the expression of fibronectin was upregulated in human PCO attached LECs and in the LECs obtained from patients with anterior polar cataracts [5, 6]. EGF has previously been demonstrated to regulate MYC and EZH2 expression [16, 17]. To explore whether MYC and EZH2 were involved in EGF inducing EMT in primary HLECs, we analyzed the expression levels of MYC mRNA and EZH2 mRNA. As shown in Figures 2(a) and 2(b), upregulation of MYC mRNA and EZH2 mRNA was detected in human PCO attached LECs compared with normal attached LECs. Furthermore, upregulation of MYC mRNA and EZH2 mRNA was detected in the LECs obtained from patients with anterior polar cataracts compared with patients with nuclear cataracts (Figures 2(c) and 2(d)).

### 3.3. EGF Induces the Expression of Myc and EZH2 in Primary HLECs

**Figure 3:**
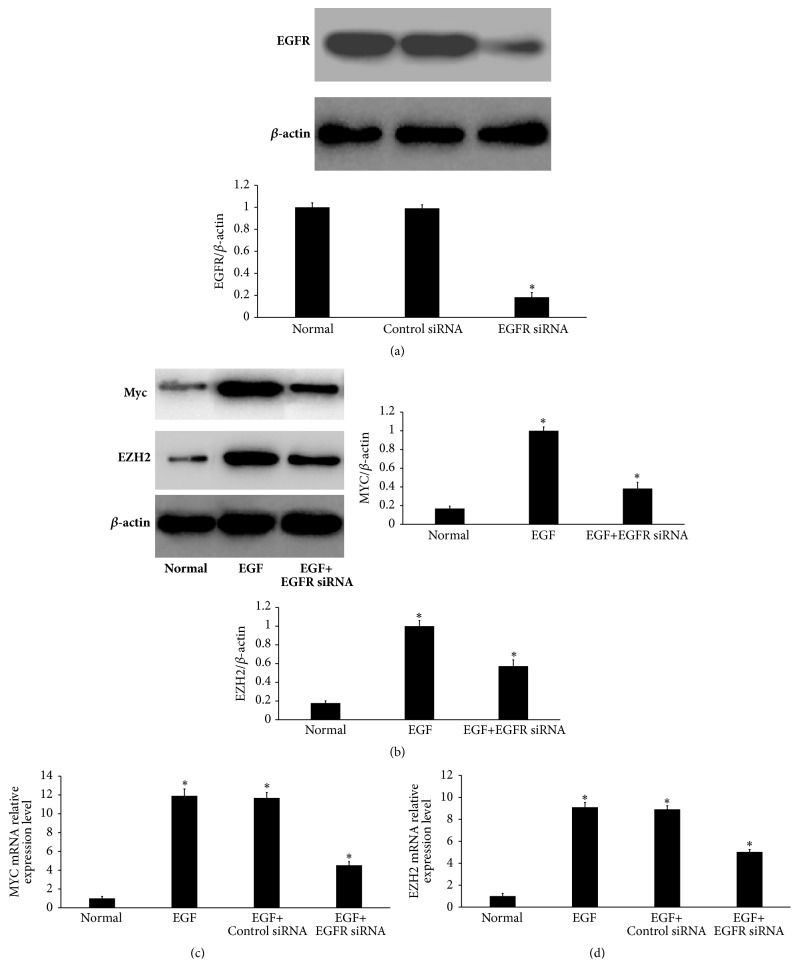
EGF induces the expression of Myc and EZH2 in primary HLECs. (a) Effects of EGFR siRNA on EGFR expression in primary HLECs. We achieved downregulation of EGFR as monitored by Western blot analysis. (b) After 48 h of EGF (10 ng/ml) treatment, MYC and EZH2 were indeed overexpressed in primary HLECs by Western blot analysis. Moreover, EGFR knockdown led to downregulation of Myc and EZH2 expression induced by EGF. ((c) and (d)) After 48 h of EGF (10 ng/ml) treatment, MYC mRNA (c) and EZH2 mRNA (d) were overexpressed as monitored by qRT-PCR. ^*∗*^*P* < 0.05 compared with normal.

It has been shown that Myc and EZH2 are overexpressed in human PCO attached LECs and LECs obtained from patients with anterior polar cataracts, indicating that MYC and EZH2 were involved in the development of PCO. We prepared to explore whether EGF regulates MYC and EZH2 expression in primary HLECs. We observed a reduction in EGFR by transfecting primary HLECs with siRNA specific to EGFR (Figure 3(a)). As shown in Figure 3(b), EGF (10 ng/ml) treatment for 48 h induced the expression of Myc and EZH2 in primary HLECs by Western blot. In addition, EGFR knockdown led to downregulation of Myc and EZH2 expression from primary HLECs induced by EGF (Figure 3(b)). Moreover, elevated MYC mRNA expression (Figure 3(c)) and upregulated EZH2 mRNA expression (Figure 3(d)) were induced by EGF. These data indicate that EGF may induce the overexpression of Myc and EZH2 in primary HLECs by activating EGFR.

### 3.4. Myc Regulates EZH2 mRNA and Protein Expression in Primary HLECs

**Figure 4:**
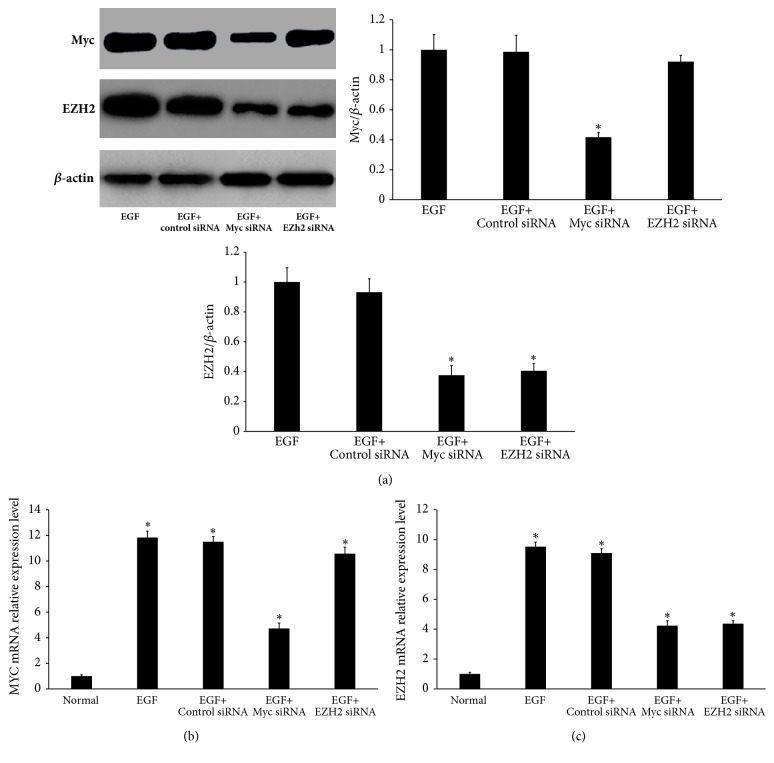
EGF can activate EZH2 transcription by activating Myc in primary HLECs. (a) 24 h after Myc or EZH2 knockdown, we measured the expression of Myc and EZH2 protein by Western blot analysis. ((b) and (c)) Primary HLECs were preincubated with Myc-specific siRNAs or EZH2-specific siRNAs for 24 hours before incubation with 10 ng/ml EGF for 48 hours. Myc mRNA and EZH2 mRNA were measured by qRT-PCR. ^*∗*^*P* < 0.05 compared with EGF (a). ^*∗*^*P* < 0.05 compared with normal ((b) and (c)).

To explore the possible involvement of EZH2 protein and mRNA expression in EGF-treated primary HLECs, primary HLECs were pretreated with siRNA-Myc and siRNA-EZH2. We observed that Myc knockdown led to downregulation of Myc and EZH2 expression induced by EGF; however, pretreatment with EZH2 siRNA only led to downregulation of EZH2 expression and did not reduce EGF-induced Myc protein expression induced by EGF (Figure 4(a)). Additionally, Myc knockdown led to downregulation of Myc mRNA; however, EZH2 knockdown did not reduce expression of Myc mRNA induced by EGF (Figure 4(b)). In addition, both Myc knockdown and EZH2 knockdown reduced expression of Myc mRNA induced by EGF (Figure 4(c)). These data indicate that EGF can indeed activate EZH2 transcription by activating Myc in primary HLECs.

### 3.5. EGF Regulates miR-26b Expression in Primary HLECs

**Figure 5:**
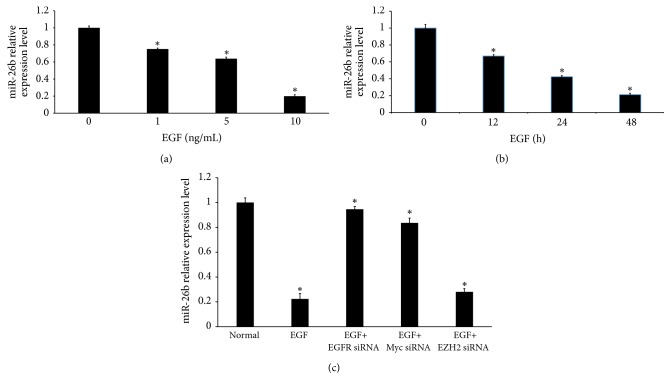
EGF regulates miR-26b expression in primary HLECs. ((a) and (b)) miR-26b expression in primary HLECs was measured by qRT-PCR. EGF stimulation inhibited the expression of miR-26b in primary HLECs. (a) The primary HLECs were incubated with the indicated concentration of EGF for 48 hours. (b) The primary HLECs were incubated with 10 ng/ml EGF for the indicated time. EGF treatment decreased the expression of miR-26b in a dose- and time-dependent manner. (c) Primary HLECs were preincubated with EGFR-specific siRNAs, Myc-specific siRNAs, or EZH2-specific siRNAs for 24 hours before incubation with 10 ng/ml EGF for 48 hours. miR-26b expression in primary HLECs was measured by qRT-PCR. ^*∗*^*P* < 0.05 compared with group without EGF.

MicroRNAs (miRNAs) can regulate the posttranscriptional expression of protein-coding mRNAs [5, 6, 11, 12]. Mechanistically, miRNAs can block translation through binding to the 3′-untranslated regions (UTRs) of target mRNAs [5, 6, 11, 12]. Increasing evidence indicates that some miRNAs play a role in the pathogenesis of PCO [5, 6, 18]. Our previous study has demonstrated that miR-26b can inhibit the proliferation, migration, and EMT of LECs by targeting Smad4 and COX-2 [6]. To further analyze whether EGF affects the expression of miR-26b in primary HLECs, we measured the expression of miR-26b by qRT-PCR. EGF significantly downregulated miR-26b in primary HLECs in a dose-dependent manner and a time-dependent manner (Figures 5(a) and 5(b)). Next, by using siRNA-mediated knockdown to deplete EGFR, Myc, or EZH2, we found that miR-26b expression suppressed by EGF was attenuated in primary HLECs pretreated with EGFR siRNA and Myc siRNA, whereas incubation with EZH2 siRNA had no effect (Figure 5(c)). Thus, miR-26b expression is consistently suppressed by EGF and the inhibitory effect is controlled by Myc.

### 3.6. miR-26b Expression Is Suppressed by EGF and the Inhibitory Effect Is Controlled by Histone Deacetylases

**Figure 6:**
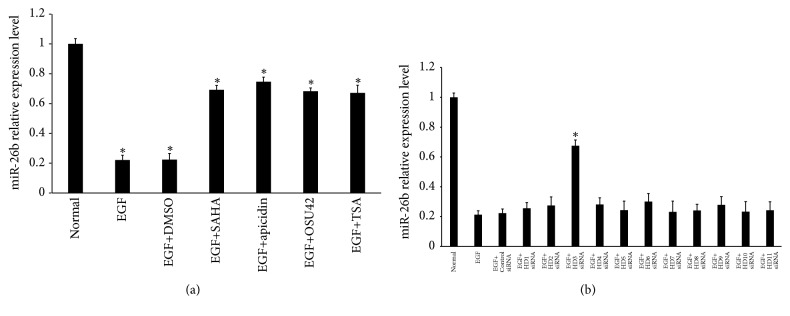
miR-26b expression suppressed by EGF is controlled by HDAC3 in primary HLECs. (a) Suberoylanilide hydroxamic acid (10 *μ*mol/L), apicidin (3 *μ*mol/L), OSU42 (2.5 *μ*mol/L), or TSA (0.4 *μ*mol/L) significantly attenuated the expression of miR-26b in primary HLECs suppressed by EGF. miR-26b expression in primary HLECs was measured by qRT-PCR. (b) Primary HLECs were preincubated with HDAC1~11-specific siRNAs for 24 hours before incubation with 10 ng/ml EGF for 48 hours. miR-26b expression in primary HLECs was measured by qRT-PCR. ^*∗*^*P* < 0.05 compared with normal (a). ^*∗*^*P* < 0.05 compared with EGF (b).

Because miRNA expression can be regulated by epigenetic histone acetylation [11, 12, 19, 20], we explored whether small molecule histone deacetylase inhibitors (HDACi) can rescue miR-26b expression in primary HLECs suppressed by EGF. We found that suberoylanilide hydroxamic acid, apicidin, OSU42, or trichostatin A (TSA) significantly attenuated the expression of miR-26b in primary HLECs suppressed by EGF (Figure 6(a)). In addition, Myc was previously reported to recruit HDAC3 to suppress expression of miRNAs [21]. Next, to elucidate the functional role of individual HDACs, primary HLECs were pretreated with siRNA-HDAC1~11. As shown in Figure 6(b), knockdown of HDAC3 partially reversed the repression of miR-26b by EGF in primary HLECs. Taken together, our data suggest that miR-26b expression suppressed by EGF is controlled by HDAC3.

### 3.7. miR-26b Downregulates EZH2 Expression by Directly Targeting Its 3′-UTR in HLECs

**Figure 7:**
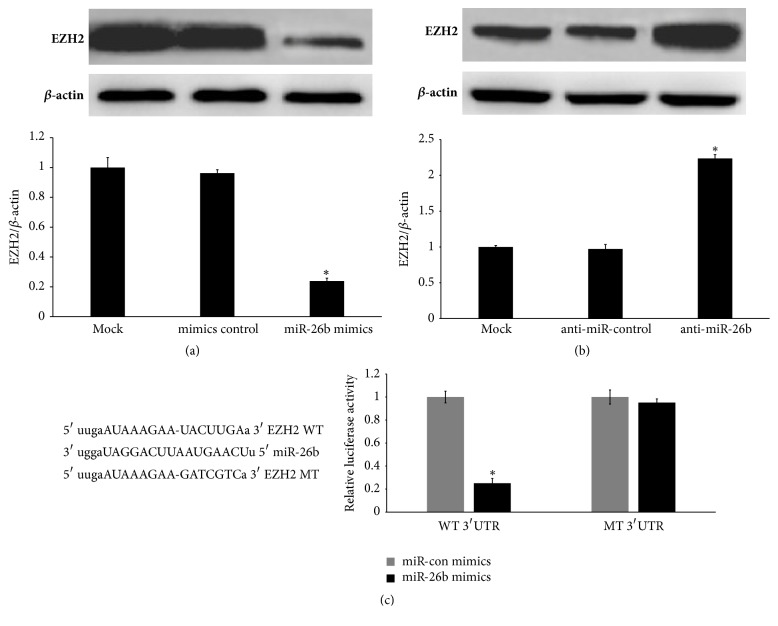
miR-26b directly controls EZH2 expression in HLECs. (a) EZH2 and *β*-actin were detected in primary HLECs after transfection with miR-26b mimics or miR-26b mimics control for 6 h by Western blot analysis. (b) Western blotting for EZH2 and *β*-actin in primary HLECs after transfection with anti-miR-26b or anti-miR-control for 6 h. (c) The luciferase reporter assays in the SRA01/04 cell line were measured. The miR-26b mimics or miR-26b mimics controls were cotransfected into plasmids with wild-type or mutant EZH2 3′-UTRs. ^*∗*^*P* < 0.05 compared with Mock ((a) and (b)). ^*∗*^*P* < 0.05 compared with miR-con mimics (c).

In primary HLECs following treatment with EGF, miR-26b was downregulated, indicating that it participated in EGF-induced EMT in HLECs. Western blot analysis showed that overexpression of miR-26b downregulated the expression of EZH2 in primary HLECs (Figure 7(a)). Next, we found that the downregulated expression of miR-26b increased the expression of EZH2 in primary HLECs (Figure 7(b)). To further explore the mechanism through which miR-26b downregulated the expression of EZH2, we used miRanda to search for 3′-UTR sequences of mRNAs encoding EZH2. We found that EZH2 mRNA contained a seed sequence for miR-26b, which suggests that miR-26b binds directly to its 3′-UTR (Figure 7(c)). In addition, luciferase reporter assays demonstrated that miR-26b directly targets EZH2 in HLECs (Figure 7(c)).

### 3.8. EGF Induces the Expression of EMT Markers in Primary HLECs via a miR-26b–Dependent Mechanism

**Figure 8:**
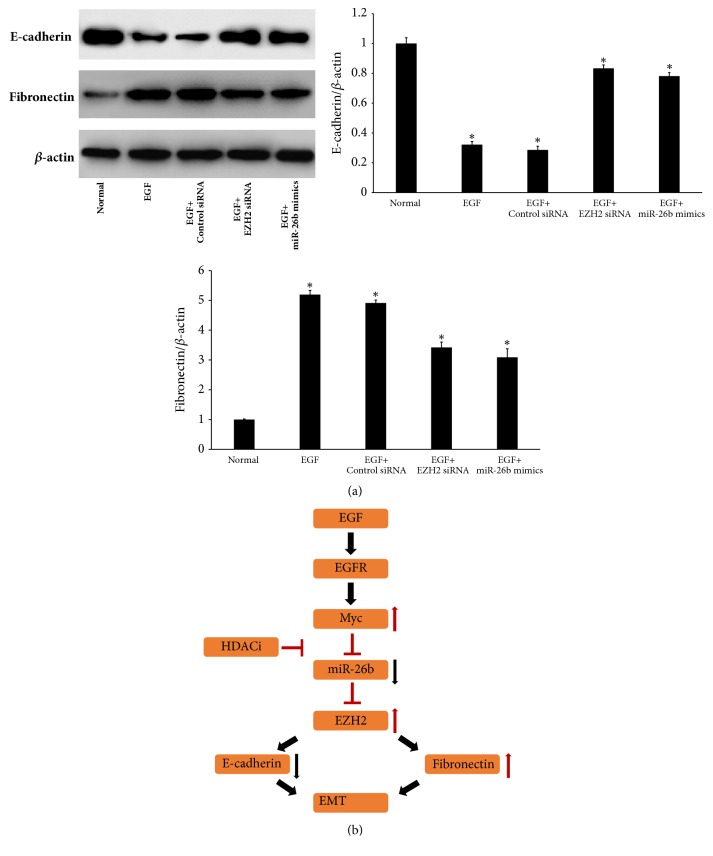
EGF induces the expression of EMT markers in primary HLECs via a miR-26b-dependent mechanism. (a) Primary HLECs were preincubated with EZH2-specific siRNAs for 24 hours or miR-26b mimics for 6 h before incubation with 10 ng/ml EGF for 48 hours. E-cadherin and fibronectin protein were measured by Western blot analysis. (b) EGF induces the expression of EMT markers in primary HLECs via a miR-26b-dependent mechanism. ^*∗*^*P* < 0.05 compared with normal (a).

To further elucidate whether miR-26b is involved in EGF inducing the expression of EMT markers in primary HLECs by directly controlling EZH2, we transfected EZH2 siRNA or miR-26b mimics into HLECs. As shown by Western blot analysis (Figure 8(a)), downregulation of E-cadherin and upregulation of fibronectin induced by EGF were attenuated by overexpressing miR-26b. Finally, Figure 8(b) illustrates the pathologic pathways associated with EGF inducing the expression of EMT markers in primary HLECs via a miR-26b-dependent mechanism.

## 4. Discussion

Earlier studies have shown that the repression of EGFR can effectively prevent the progression of PCO by inhibition of LECs proliferation [3]. Except for LECs proliferation, EMT of LECs is also considered to be a central role in the pathogenesis of PCO. Recently, EGF has been associated with the initiation of EMT in many cancers [9, 22]. Our data clearly showed that EGF signaling can regulate EMT of LECs. EGF and EGFR signaling induced the expression of Myc in the LECs. Myc overexpression inhibited miR-26b by recruitment of HDAC3, which in turn induced the expression of EZH2 and promotes the progression of EMT.

The protooncogene Myc, also named c-Myc and bHLH transcription factor, activates cell cycle progression. Downregulation of Myc could significantly inhibit LECs proliferation and induce cell apoptosis [23]. Additionally, Myc mediates cancer cell EMT progression in various types of cancer [24, 25]. Recently, the studies showed that EGF contributed to the initiation of EMT via activating Myc in many cancers [22]. Our data demonstrated that upregulation of MYC mRNA was detected in human PCO attached LECs and the LECs obtained from patients with anterior polar cataracts, which indicated that MYC was involved in the development of PCO. In addition, the present data showed that EGF may induce the overexpression of Myc in primary HLECs by activating EGFR. These data indicated that EGF can indeed be involved in the development of PCO by activating Myc in primary HLECs.

EZH2 is a histone lysine methyltransferase and the catalytic subunit of the polycomb repressor complex 2 (PRC2). It catalyzes trimethylation of lysine 27 of histone H3 (H3K27me3) to repress gene transcription. Our data showed that the expression levels of EZH2 mRNA are increased in human PCO attached LECs and LECs obtained from patients with anterior polar cataracts, which indicated that EZH2 was involved in the development of PCO. Additionally, Koh CM et al. have shown that MYC can regulate EZH2 through transcriptional and posttranscriptional means [26]. Consistent with the previous study, our data indicated that EGF can activate EZH2 transcription by activating Myc in primary HLECs [26–28].

MicroRNAs (miRNAs) can regulate the posttranscriptional expression of protein-coding mRNAs [5, 6, 11, 12]. There is growing evidence of miRNAs playing a role in the pathogenesis of PCO [5, 6, 18]. Our previous study has shown that miR-26b can inhibit the proliferation, migration, and EMT of LECs [6]. In the present study, EGF significantly inhibited the expression of miR-26b in primary HLECs in a dose-dependent manner and a time-dependent manner. Histone modifications have been demonstrated to be important in the pathogenesis of PCO by inhibiting EMT of LECs [29, 30]. Increasing evidence illustrates that miRNA expression can be regulated by epigenetic histone acetylation [11, 12, 19, 20]. Our studies showed that small molecule histone deacetylase inhibitors (HDACi) can rescue miR-26b expression in primary HLECs suppressed by EGF, consistent with the previous study [31]. Mechanistically, our data suggest that miR-26b expression suppressed by EGF is controlled by HDAC3.

MiR-26b has been reported to suppress tumorigenesis by binding to the 3′-UTRs of EZH2 in several tumors [26, 32]. In the present study, we identified EZH2 as a direct target of miR-26b in LECs and found that suppression of EZH2 by miR-26b is necessary for expression of EMT markers induced by EGF in primary HLECs.

## 5. Conclusion

In summary, this study reveals a new mechanism in which EGF and EGFR signaling induces the progression of EMT via a miR-26b-dependent mechanism in the LECs. These findings provide novel evidence that EGF and EGFR signaling induced the expression of Myc. Then, Myc overexpression inhibited miR-26b by recruitment of HDAC3, which in turn induced the expression of EZH2 and promoted the progression of EMT. Thus, these results also indicated that EGF/EGFR is a potential therapeutic target for the treatment of PCO.

## Figures and Tables

**Table 1:** Primers used for qRT-PCR and cloning.

Primers	Sequence	
	Sense	Antisense
GAPDH	5′-AGGTCGGTGTGAACGGATTTG-3′	5′-TGTAGACCATGTAGTTGAGGTCA-3′
U6	5′-CTCGCTTCGGCAGCACA-3′	5′-AACGCTTCACGAATTTGCGT-3′
miR-26b	5′-CTCAAGGGCTTGTGCTGACT-3′	5′-ACCTCAGCCTAGTGCAGGAA-3′
Fibronectin	5′-TCTGTGCCTCCTATCTATGTGC-3′	5′-GAGGGACCACGACAACTCTTC-3′
*α*-SMA	5′-CCGACCGAATGCAGAAGGA-3′	5′-ACAGAGTATTTGCGCTCCGAA-3′
Myc	5′-TCAGAGAAGCTGGCCTCCTA-3′	5′-TCGTTGGAGGAGAGCAGAGA-3′
EZH2	5′-CCAACATTGGAGTGATTCAG-3′	5′-TCATCAGATGATTTAGCCCA-3′
HDAC1	5′-GCGAGCAAGATGGCGCAGACT-3′	5′-GTGAGGCTTCATTGGGTGCCCT-3′
HDAC2	5′-TGAAGCCAAACTTCCTCAAACA-3′	5′-CGGAGGACAAGAGGACAGATG-3′
HDAC3	5′-CACATCGTCATCTCGATTTCCT-3′	5′-GGCATGGCTCTCTGAAACCTTA-3′
HDAC4	5′-GAGTACTGCCGAGCAGTCTGAA-3′	5′-CACTTTAATTGGGTCTGGAGGC-3′
HDAC5	5′-TTCTTCAACTCCGTAGCC-3′	5′-TCCCATTGTCGTAGCG-3′
HDAC6	5′-TGTGGCTGCCCGCTATGCAC-3′	5′-GGGGCCAGAACCGACCATGC-3′
HDAC7	5′-ACCCAACCTCAATGCC-3′	5′-GATGCCAACGGAAAGG-3′
HDAC8	5′-CCAGCCACAGAAGGGATA-3′	5′-TTCCGTCGCAATCGTAAT-3′
HDAC9	5′-GTCCCTGCCCAATATCAC-3′	5′-GCTGTTCGGTTTGCCCTC-3′
HDAC10	5′-CCGGCAGAGGGCGTGTTGAG-3′	5′-CAAGGCAGCTGTCAGGCGCT-3′
HDAC11	5′-ACAACCGCCACATCTAC-3′	5′-AGGGACCTCCTCACATT-3′
EZH2-3′UTR-WT-luc	5′-TATCTAGACATCTGCTACCTCCTCCC-3′	5′-ATGCGGCCGCGATTCAACAAGGACAA-3′
EZH2-3′UTR-MT-luc	5′-TATCTAGACATCTGCTACCTCCTCCC-3′	5′- CTGCTACCTCCTCCC-3′

## Data Availability

All relevant data used to support the findings of this study are included within the article.
